# Weight and Glycemic Control Outcomes of Bariatric Surgery and Pharmacotherapy in Patients With Melanocortin-4 Receptor Deficiency

**DOI:** 10.3389/fendo.2021.792354

**Published:** 2022-01-13

**Authors:** Esphie Grace Fodra Fojas, Saradalekshmi Koramannil Radha, Tomader Ali, Evan P. Nadler, Nader Lessan

**Affiliations:** ^1^ Research Institute, Imperial College London Diabetes Centre, Abu Dhabi, United Arab Emirates; ^2^ Division of Pediatric Surgery, Children’s National Hospital, Washington, DC, United States

**Keywords:** bariatric surgery, MC4R deficiency, monogenic obesity, obesity treatment, obesity pharmacotherapy, metabolic surgery

## Abstract

**Background:**

Melanocortin-4 receptor (*MC4R*) mutations are the most common of the rare monogenic forms of obesity. However, the efficacy of bariatric surgery (BS) and pharmacotherapy on weight and glycemic control in individuals with MC4R deficiency (MC4R-d) is not well-established. We investigated and compared the outcomes of BS and pharmacotherapy in patients with and without MC4R-d.

**Methods:**

Pertinent details were derived from the electronic database among identified patients who had BS with MC4R-d (study group, SG) and wild-type controls (age- and sex-matched control group, CG). Short- and long-term outcomes were reported for the SG. Short-term outcomes were compared between the two groups.

**Results:**

Seventy patients were screened for MC4R-d. The SG [six individuals (four females, two males); 18 (10–27) years old at BS; 50.3 (41.8–61.9) kg/m^2^ at BS, three patients with homozygous T162I mutations, two patients with heterozygous T162I mutations, and one patient with heterozygous I170V mutation] had a follow-up duration of up to 10 years. Weight loss, which varied depending on mutation type [17.99 (6.10–22.54) %] was stable for 6 months; heterogeneity of results was observed thereafter. BS was found superior to liraglutide on weight and glycemic control outcomes. At a median follow-up of 6 months, no significant difference was observed on weight loss (20.8% vs. 23.0%, *p* = 0.65) between the SG and the CG [eight individuals (four females, four males); 19.0 (17.8–36.8) years old at BS, 46.2 (42.0–48.3) kg/m^2^ at BS or phamacotherapeutic intervention]. Glycemic control in patients with MC4R-d and Type 2 diabetes improved post-BS.

**Conclusion:**

Our data indicate efficacious short-term but varied long-term weight loss and glycemic control outcomes of BS on patients with MC4R-d, suggesting the importance of ongoing monitoring and complementary therapeutic interventions.

## Introduction

Obesity etiology is complex with interplay between genetic and environmental factors. Early twin studies indicate that 40%–70% of obesity is hereditary (1). “Simple obesity” is mostly a polygenic disorder. However, several, albeit rare, monogenic forms are recognized. The commonly reported genetic dysregulations include mutations in the leptin and receptor (*LEP/LEPR*), proopiomelanocortin (*POMC*), proprotein convertase subtilisin/kexin type 1 (*PCSK1*) and melanocortin-4 receptor (*MC4R*) genes (2, 3).

Of those genes, the most common monogenic mutation with a prevalence of up to 6% have been reported to be alterations in the *MC4R* alleles (4–6). Melanocortin-4 receptor (MC4R) is an integral part of the leptin–melanocortin pathway, which is responsible for central energy homeostasis and body weight regulation (7). MC4R is a transmembrane G-protein-coupled receptor expressed in the hypothalamic neurons and follows an autosomal dominant mode of inheritance. Phenotypic characteristics in individuals with homozygous and also heterozygous *MC4R* mutation include excessive hunger and hyperphagia, which is the main driver of the significant and rapid weight gain (8, 9). As this is a genetic effect, it carries a substantial behavioral impact, especially in feeding and satiety (10, 11). In the UK, a recent study has estimated the frequency of heterozygous *MC4R* loss-of-function mutations at 0.30%, a considerably higher rate compared to previous approximations (12).

Treatment of obesity, in general, is a major challenge. Lifestyle interventions and pharmacotherapy may lead to some weight loss, but relapse is common (13, 14). Bariatric surgery (BS) is currently the most efficacious treatment for obesity with good short-term and long-term outcomes (15). However even when BS is part of the treatment algorithm for cases of “simple obesity”, there is risk of weight regain in the long term (16).

Treatment of monogenic obesity (MO) may be even more challenging as MO is rare, and data on efficacy of different interventions are also sparse. Failure to lose sufficient weight or weight regain may be more common in patients with MO as their biological drive to eat may be unaffected by the standard interventions, including BS. Previous studies published in 2011 and 2014 suggest that patients with heterozygosity of the *MC4R* gene can be treated effectively in the short term with BS, but the longer-term success rates are not well described (17, 18). Similar observation in a patient with homozygous MC4R deficiency (MC4R-d) was reported in 2015 (19). A systematic review in 2021 synthesized outcomes of BS among patients with monogenic forms of obesity and found that weight loss results were inconsistent for patients with MC4R-d (20). Our group has previously reported short-term effects of BS in three patients with homozygous *MC4R* mutations, the youngest of whom was only 4 years old when a laparoscopic vertical sleeve gastrectomy (LSG) was performed (21). Here, we report longer-term BS efficacy results up to 10 years in these patients with homozygous *MC4R* mutation with a novel report on revisional BS long-term outcomes in a sibling pair. Three additional cases of heterozygous *MC4R* mutations are presented, and pharmacotherapeutic outcomes are also discussed.

## Materials and Methods

**Figure 1:**
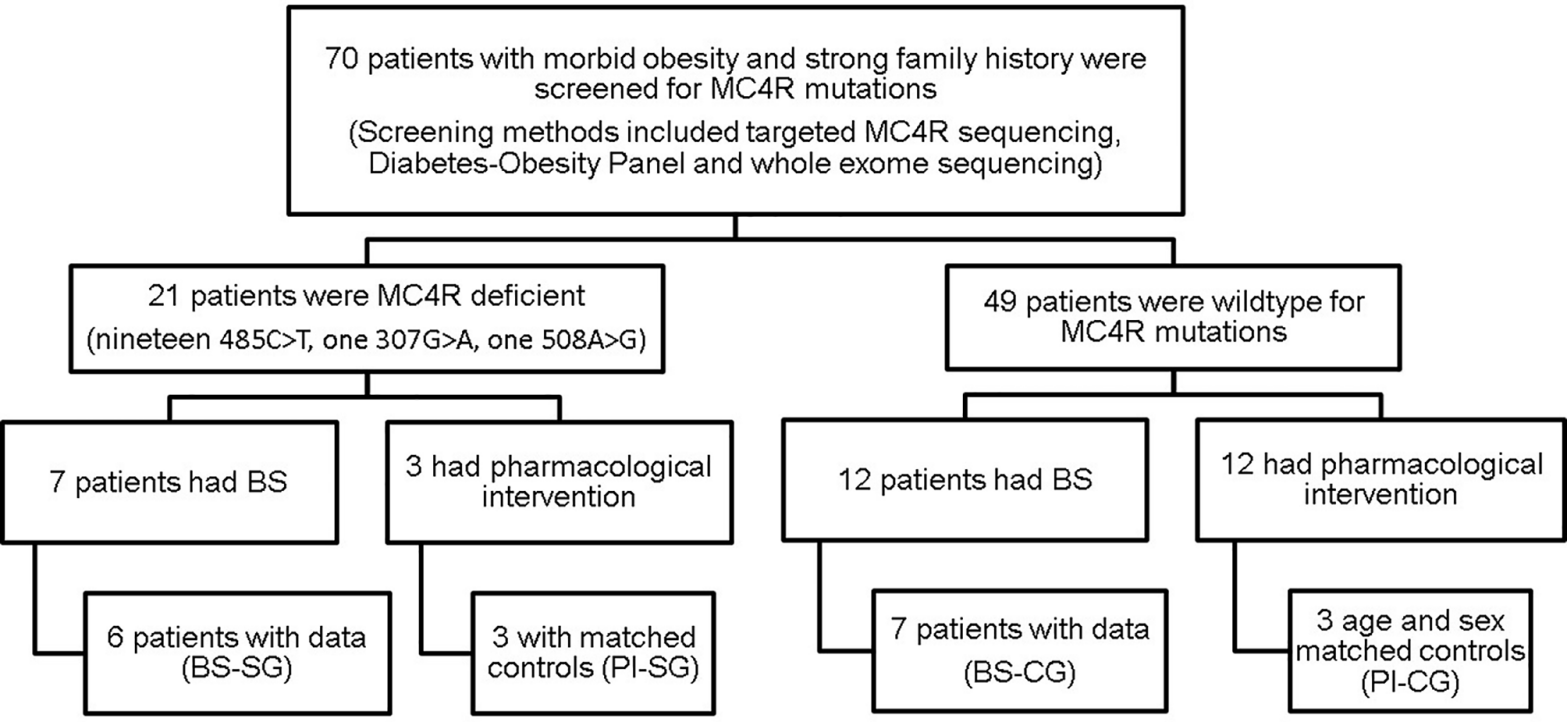
Flowchart of study population: an overview of patient selection. MC4R, melanocortin-4 receptor; BS, bariatric surgery; PI, pharmacological intervention; SG, study group; CG, control group.

Patients who have had genetic screening were identified from the Imperial College London Diabetes Centre (ICLDC) electronic database. Genetic screening is performed in patients suspected to have a genetic component for obesity such as morbid obesity and strong family history. *MC4R* mutations were screened through either targeted sequencing for *MC4R* gene, whole exome sequencing, or Diabetes-Obesity New Generation Sequencing panel. Patients who tested positive for MC4R-d (study group, SG) and age- and sex-matched individuals with normal *MC4R* genotype (control group, CG) were identified based on the reported results from the genetic screening. The study flow diagram is presented in **Figure 1**.

Case history and pertinent details such as age, weight, and HbA1c before and after BS and pharmacotherapy were derived from electronic medical records. Weight was measured and recorded as part of vitals assessment. HbA1c test was performed in the in-house laboratory. Medication compliance was assessed based on physicians’ or clinic notes. Patients’ glycemic control was managed at ICDLC. BS was performed in different centers locally and abroad. For patients who had multiple or revisional BS, the last surgery was used for pertinent analyses. Data were analyzed and presented as median (interquartile range—IQR).

Written informed consent for using medical data for research purpose in an anonymized form was obtained from all participants at the time of first visit. The study was approved by the Research Ethics Committee at ICLDC and followed the Declaration of Helsinki, 1996.

## Results

Genetic screening for *MC4R* mutation(s) was performed on 70 ICLDC patients between 2010 and 2020. Twenty-one patients were tested positive to have a mutation in *MC4R* gene. Three mutations were identified: c.485C>T (T162I), 508A>G (I170V), and 307G>A (V103I). Six individuals composed the SG [four females, two males; 18 (10–27) years old at BS; 50.3 (41.8–61.9) kg/m^2^ at BS; three patients with homozygous T162I mutations, two patients with heterozygous T162I mutations, and one patient with heterozygous I170V mutation]. Eight individuals composed the CG [four females, four males; 19.0 (17.8–36.8) years old at BS, 46.2 (42.0–48.3) kg/m^2^ at BS or phamacotherapeutic intervention]. All patients are Emirati.

### Patients With MC4R-d: Presentation of Cases

Patient 1 (P1) was born full term normal delivery (FTND) with a birthweight of 3.5 kg to non-consanguineous parents. He started gaining weight around 3 years of age. He weighed 143 kg (BMI 52 kg/m^2^; >99^th^ percentile) at the age of 12 years. He underwent LSG in another center in the UAE before a genetic diagnosis in our clinic was made. He lost 18 kg in 6 months and his glycemic control improved (HbA1c 6.1%, off metformin). He started to regain his lost weight, reaching a weight of 177 kg (BMI 60 kg/m^2^) by 6 years post-surgery. A second operation, RYGB was performed at 18 years of age. He lost 15 kg (BMI 55 kg/m^2^) in 1 year and had an HbA1c of 5.6% off metformin. In the recent 2 years, he was able to maintain good glycemic control (mean HbA1c 5.5%; range 5.4%–5.7%). However, his weight fluctuated; mean BMI was 52.7 kg/m^2^ (range 50.0–56.8 kg/m^2^). Genetic diagnosis was available some months after the operation and showed him to be homozygous for the missense mutation T162I in the *MC4R* allele.

Patient 2 (P2) is the older sister of P1. She was also born FTND with a birth weight of 3.5 kg. She started gaining weight quite early, around the age of 1 year. She weighed 150 kg (BMI 60 kg/m^2^; >99^th^ percentile) at the age of 14; glycemic control was poor with an HbA1c of 9.7%. She underwent LSG, also prior to genetic diagnosis. She lost 22 kg (BMI 53 kg/m^2^) in 6 months. Her weight was consistently lower postoperatively with slight fluctuations near 130 kg, but her HbA1c varied widely. She underwent a second operation, RYGB, at the age of 20 years. She lost 9 kg (BMI 47 kg/m^2^) by 3 months post-RYGB and maintained her weight loss up to 2 years later; glycemic control did improve, but remained inadequate (HbA1c 7.4%). In the subsequent 2 years, there were slight variations in her BMI (mean 46.0; range 43.8–48.2 kg/m^2^) and wider fluctuations in her glycemic control (mean 8.4%; range 6.9%–9.6%). Most recently, she has been on a diet program and has managed to lose 13 kg in weight, with good improvement in her glycemic control (HbA1c dropping from 9.0% to 5.2%) in a matter of 2 months. She is currently on vidagliptin + metformin. Genetic test results available after LSG have also shown a genotype of *MC4R*-/- with the missense mutation T162I.

Patient 3 (P3) was born full term to consanguineous Emirati parents (second cousins). Weight gain and obesity started at 6 months of age. At the age of 10 months, she was unable to sit normally and was hospitalized to help manage her excessive weight. She had acanthosis nigricans, impaired fasting glycemia, elevated fasting insulin, and HOMA-IR. Other features of note were severe vitamin D deficiency, hypertension, and obstructive sleep apnoea (OSA). At the age of 4, she weighed 67.8 kg (BMI 44 kg/m^2^). She had a full assessment including a review of management options by the Hospital Ethics Committee and underwent LSG in the United States. Four months post-surgery, her BMI was 9 points lower; she had normal fasting glucose, HbA1c, vitamin D, and cortisol. She lost over 13 kg (BMI 33 kg/m^2^) 9 months after sleeve gastrectomy. However, she subsequently gained 2 kg within 2 months. By year 4 post-operatively, her BMI was 35.8 kg/m^2^ with an HbA1c of 6.0%. One year later (5 years after her surgery), her BMI was 39.3 kg/m^2^, close to her pre-operative BMI; HbA1c was 6.3% and she was prescribed metformin. At 6.5 years post-BS, her BMI is 42.1 kg/m^2^. Her genetic testing (done at age 4) showed her to be homozygous for an *MC4R* mutation (T162I). P3 has a younger brother with the same *MC4R* mutation who recently had BS at 5 years of age and presented with non-alcoholic steatohepatitis (stage 3–4), a condition not previously seen in P3. P3 also has two other siblings with *MC4R* null genotype and rare *ACBD5* mutation, which affects peroxisomal oxidation of very long chain fatty acids.

Patient 4 (P4), aunt of P3, started to become overweight at age 15 years. She had impaired fasting glucose (IFG) with normal HbA1c levels. Other history of relevance included a strong familial history of diabetes and hyperlipidemia. She was able to lose 20 kg of weight through diet but regained this same amount. At age 33 years, she was severely obese (weight 114 kg, BMI 49 kg/m^2^). She underwent sleeve gastrectomy and had lost 20 kg (BMI 40 kg/m^2^) in 4 months. Over a year after surgery, her BMI was 32.9 kg/m^2^ (total weight loss of approximately 27 kg) and her HbA1c was 5.4%. Two years post-BS, her BMI increased to 36.1 kg/m^2^ and her HbA1c was slightly lower at 5.1%. Genetic testing (performed and available before surgery) revealed that she was heterozygous for T162I mutation in the *MC4R* gene.

Patient 5 (P5), the sister of P4, was seen in our clinic at the age of 21 years. At the time, she weighed 181 kg (BMI 71 kg/m^2^). Comorbidities included hypothyroidism, IFG and impaired glucose tolerance (IGT), dyslipidemia, fatty liver, OSA, hypertension, and deficiencies in vitamin B12 and D. A trial of dietetic intervention and medical treatment with liraglutide, with a target BMI of lower than 60 kg/m^2^ were unfortunately unsuccessful. She proceeded with sleeve gastrectomy (pre-BS BMI 68 kg/m^2^) at the age of 24 years and had a drastic weight loss of 18 kg (BMI 60 kg/m^2^) in 1.5 months, with a further 16 kg (BMI 54 kg/m^2^) weight loss in the subsequent 2 months and a reduction in her HbA1c to 4.9%. She is currently on vitamins B12 and D3, iron, and thyroxine supplementation. Genetic testing (done pre-operatively) showed her to be heterozygous for T162I *MC4R* mutation.

Patient 6 (P6) is a 16-year-old male with a strong family history of diabetes who first came to the center for pediatric obesity management. He was noted to have acanthosis, and by age 17, he developed Type 2 diabetes (T2D). He had poor glycemic control, at least in part due to non-compliance to medication. He was also hypertensive, and had hypertriglyceridemia, proteinuria, hyperuricemia, and joint pain. By age 23, he weighed 110 kg (BMI 35 kg/m^2^) and had an HbA1c of 9.6%. He underwent sleeve gastrectomy and lost 23 kg (BMI 28 kg/m^2^) in 7 months with excellent glycemic control on metformin only. He is recently off metformin, has normal blood pressure, and is taking vitamin D3, multivitamins, and vitamin B12. *MC4R* sequencing revealed heterozygosity with variant I170V, a missense alteration.

### Weight Loss and Glycemic Control Outcomes

#### Bariatric Surgery

Patient characteristics and response to BS are summarized in **Table 1**. Genetic testing revealed two *MC4R* genotypes [five (83.3%) T162I, one (16.7%) I170V] in our cohort. LSG was the BS performed for all patients, with two siblings (P1 and P2) who had RYGB as revisional BS procedures.

**Table 1:** Patient characteristics and response to bariatric surgery procedures in patients with *MC4R* deficiency.

Patient	P1		P2		P3	P4	P5	P6
**Sex**	Male		Female		Female	Female	Female	Male
**Zygosity**	Homozygous		Homozygous		Homozygous	Heterozygous	Heterozygous	Heterozygous
**Variant**	T162I		T162I		T162I	T162I	T162I	I170V
**Type of surgery**	LSG	RYGB	LSG	RYGB	LSG	LSG	LSG	LSG
**Age (years) at surgery**	12	18	14	20	4	33	25	22
**Weight (kg) before LSG**	143	177	150	130	67.8	114	171	110
**BMI (kg/m^2^) before LSG**	52.0	60.5	60.0	50.3	44.0	49.0	67.6	35.0
**Lowest weight (kg) after LSG**	125	149	124	112	54.5	77	137.2	88.1
**Lowest BMI (kg/m^2^) after LSG**	45.4	50.7	48.6	43.8	33.3	32.9	53.6	28.1
**% Weight loss**	12.6	15.8	17.0	13.8	19.6	32.5	19.8	19.9
**HbA1c (%) before LSG**	8.1	6.1	NA	8.0	NA	NA	5	9.6
**HbA1c (%) after LSG**	5.6–6.6	5.4–5.9	6.5–11.4	6.8–9.6	5.5–6.3	4.8–5.4	4.9	5.7–8.3

LSG, laparoscopic sleeve gastrectomy; RYGB, Roux-en-Y gastric bypass; BMI, body mass index; MC4R, melanocortin-4 receptor; NA, not available.

**Figure 2:**
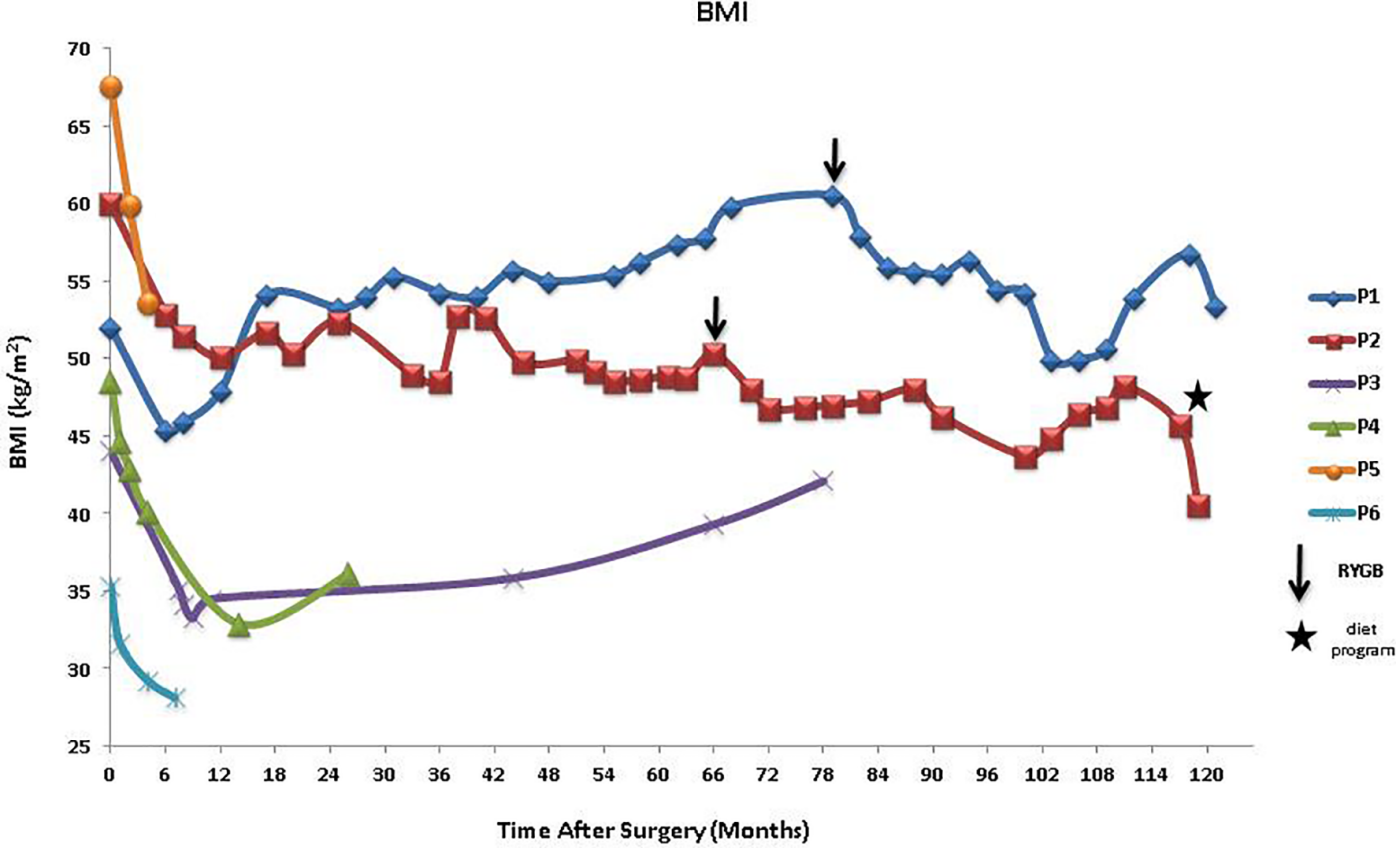
BMI after LSG in patients with MC4R deficiency showing other interventions, RYGB, and diet program. BMI, body mass index; LSG, laparoscopic sleeve gastrectomy; RYGB, Roux-en-Y gastric bypass; MC4R, melanocortin-4 receptor.

For all six patients, stable weight loss was observed up to at least 6 months (**Figure 2**), supporting our former observation that MC4R may not be essential for short-term weight loss post-BS. However, our data indicate heterogeneity in response to LSG after 6 months: one patient had continued net weight loss for over 5 years (P2); two patients (P3, P4) continued to lose weight for several months with slight increases observed subsequently; while one patient (P1) regained his initial weight after over a year, with further weight gain until 6.5 years when he had a revisional RYGB procedure. Outcomes for P1 and P2, who are siblings with the same mutation and second BS (Roux-en-Y gastric bypass), were very different.

**Figure 3:**
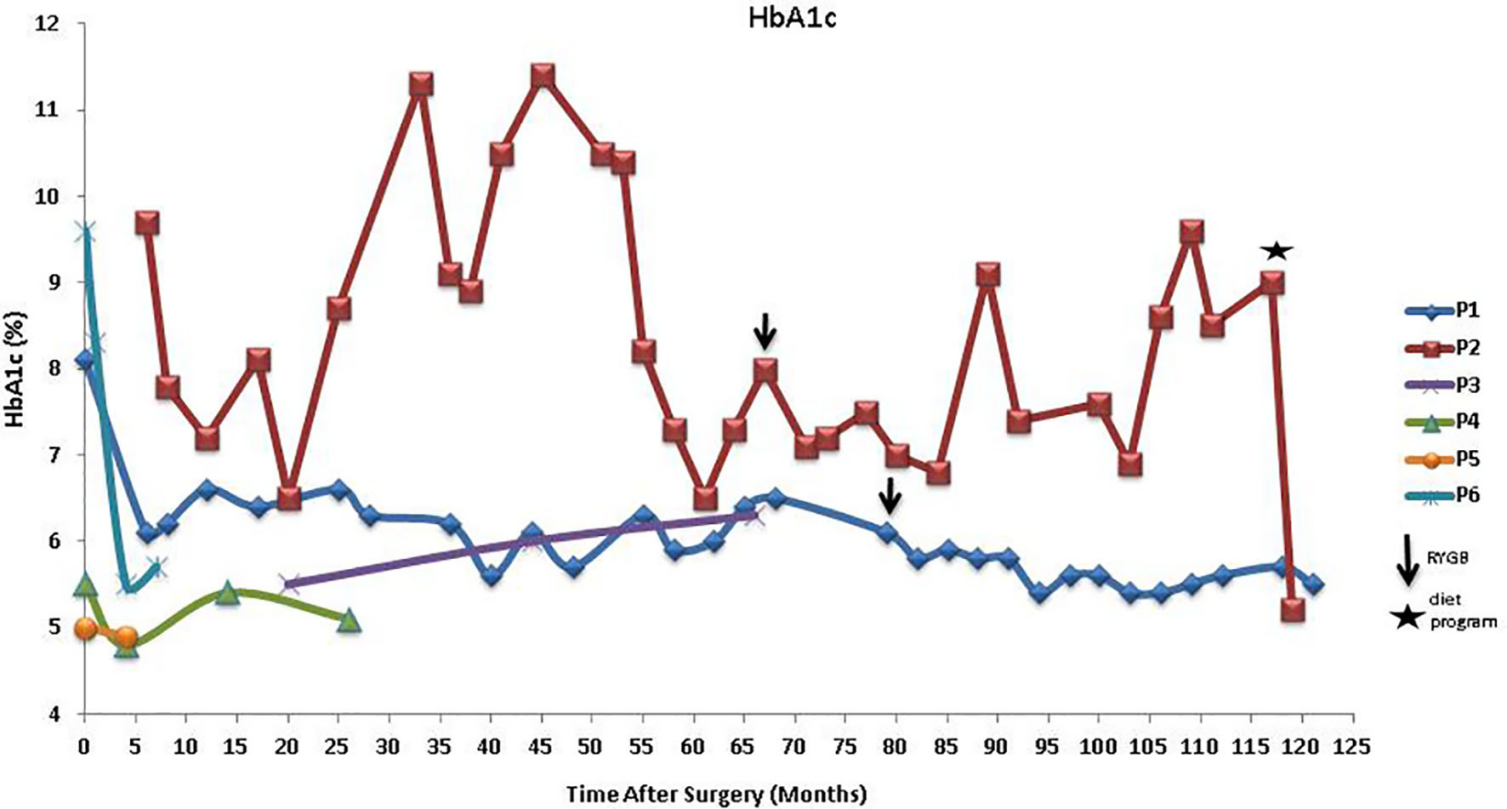
HbA1c after LSG in patients with MC4R deficiency showing other interventions, RYGB, and diet program. LSG, laparoscopic sleeve gastrectomy; RYGB, Roux-en-Y gastric bypass; MC4R, melanocortin-4 receptor.

Glycemic control outcomes of the patients after BS are also dissimilar (**Figure 3**). Although P1 has increasingly gained weight post-LSG, his HbA1c levels have lessened and were consistently lower than his pre-surgery HbA1c. In contrast, his sister, P2, who had significant weight reduction post-LSG and RYGB has not seen improvement in her glycemic control; her HbA1c levels were widely fluctuating and were at times much higher than her pre-operative values. Interestingly, her recent diet program (self-reported as composed of low-calorie diet, calorie counting, and no addition of sugar whether white, brown, or artificial sweetener) made her lose 13 kg in less than 3 months, leading to a drastic drop in her HbA1c (from 9.0% to 5.2%). Similar trends were observed for P3 who had a slight increase in HbA1c and a modest weight gain, and P6 who had weight loss and a net reduction in HbA1c levels. Slight variations in glycemic control were observed with considerable weight loss for P4 and P5 who are sisters.

#### Pharmacotherapy

Our limited data (**Table 2**) indicate apparent lack of efficacy of liraglutide (1.8 mg OD) in homozygous T162I mutation in short-term use; weight gain and increased HbA1c level were observed in the female patient P2 after 3 months of treatment. In contrast, 14 months use of liraglutide (3.0 mg OD) did benefit patient P5 (heterozygous T162I deficit) with a weight loss of 5.6% (from 179 kg to 169 kg) and a 7.4% improvement (from 5.4% to 5.0% HbA1c) in glycemic control. Short-term (3 months) use of orlistat (120 mg TID) did not benefit patient P6 (heterozygous I170V) in both weight loss and glycemic control as the patient, in fact, had small weight and HbA1c increase.

**Table 2:** Patient characteristics and response to pharmacotherapy in patients with MC4R deficiency.

Patient Characteristics	Before BS
P2*	
Pharmacotherapy	Liraglutide
Dose	1.8 mg OD
Age (years) at prescription (Rx)	19
Weight (kg) before Rx	125
BMI (kg/m^2^) before Rx	48.8
Treatment duration (months)	3
Lowest weight (kg) after Rx	129
Lowest BMI (kg/m^2^) after Rx	50.3
% Weight loss	−3.1%
HbA1c (%) before Rx	7.3
HbA1c (%) after Rx	8.0
**P5**	
Pharmacotherapy	Liraglutide
Dose	3.0 mg OD
Age (years) at prescription (Rx)	22
Weight (kg) before Rx	179
BMI (kg/m^2^) before Rx	70
Treatment duration (months)	14
Lowest weight (kg) after Rx	169
Lowest BMI (kg/m^2^) after Rx	66
% Weight loss	5.6
HbA1c (%) before Rx	5.4
HbA1c (%) after Rx	5.0–5.1
**P6**	
Pharmacotherapy	Orlistat
Dose	120 mg TID
Age (years) at prescription (Rx)	21
Weight (kg) before Rx	110
BMI (kg/m^2^) before Rx	35.1
Treatment duration (months)	3
Lowest weight (kg) after Rx	112
Lowest BMI (kg/m^2^) after Rx	35.9
% Weight loss	−1.8%
HbA1c (%) before Rx	9.6
HbA1c (%) after Rx	10.2

MC4R, melanocortin-4 receptor; OD, once daily; TID, three times daily; BMI, body mass index. *Before RYGB.

Patients P2, P5, and P6 were 19, 22, and 22 years of age, respectively, when they started the treatments. Patient P2 was treated with liraglutide after LSG due to weight regain. However, due to unsatisfactory outcomes, revisional BS, RYGB, was performed. Both patients P5 and P6 who were treated with liraglutide and orlistat, respectively, were tried on these pharmacotherapeutic interventions prior to LSG. Overall, BS was found superior to liraglutide and orlistat for both weight loss and glycemic control outcomes.

### Weight Loss and Glycemic Control Comparison in Individuals With and Without MC4R-d

Weight loss percentage and glycemic control lowering at a median of 6 months post-intervention were compared between age- and sex-matched MC4R-deficient and wild-type controls (**Table 3**).

**Table 3:** Weight loss and glycemic control comparison following surgical and pharmacological interventions at median 6 months between age- and sex-matched individuals with and without *MC4R*-deficiency.

Type of intervention	Age (MC4R-deficient vs. MC4R wild type	Sex	Mutation	Zygosity	Weight loss %		HbA1c % lowering	
					*MC4R*-deficient	*MC4R* wild type	*MC4R*-deficient	*MC4R* wild type
Sleeve gastrectomy	33.5/32.2	F	T162I	Heterozygous	19.05	15.24	NA	0.5
	23/18	M	I170V	Heterozygous	22.54	29.9	3.9	0.2
	18/17.8	M	T162I	Homozygous	8.08	35.27	2	0.3
	14/18	F	T162I	Homozygous	6.91	23.75	NA	NA
	24.5/18	F	T162I	Heterozygous	20.81	23.75	-0.1	NA
Liraglutide (3 mg OD)	22.9/18.2	F	T162I	Heterozygous	4.89	5.61	0.4	0.3
Orlistat (120 mg TID)	20.5/19.5	M	I170V	Heterozygous	-0.91	10.29	NA	NA
	12/14.5	M	T162I	Homozygous	-4.62	-4.1	NA	NA

MC4R, melanocortin-4 receptor; OD, once daily; TID, three times daily; NA, not available.

**Figure 4:**
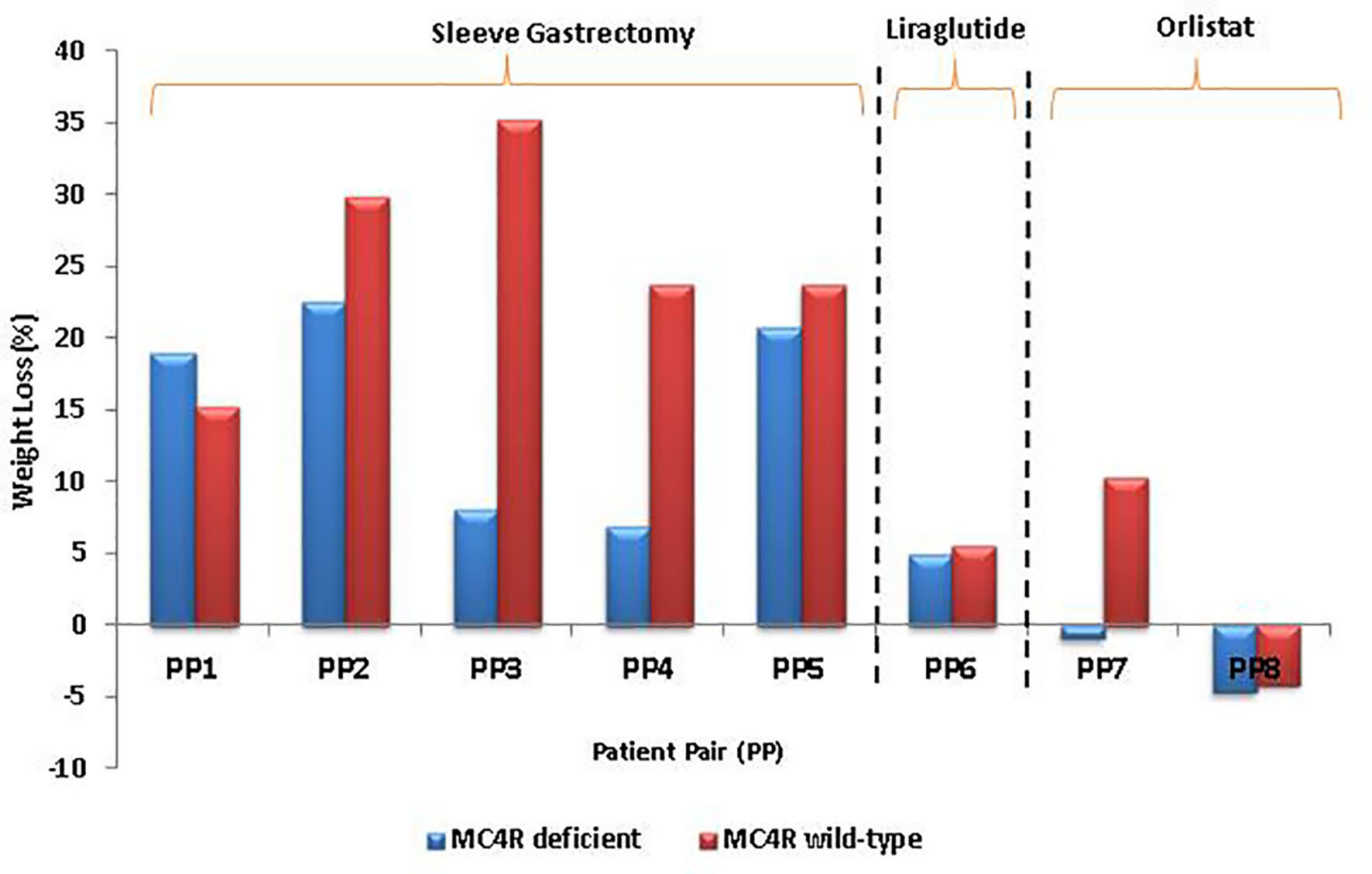
Comparison of weight loss following surgical and pharmacological interventions at median 6 months between age- and sex-matched individuals with and without MC4R-deficiency, as shown in **Table 3**. MC4R, melanocortin-4 receptor.

Weight loss at median 6 months post BS was not significantly different between patients with and without *MC4R*-d (*p* = 0.65). However, the weight loss post BS in patients with *MC4R*-d showed variation depending on the type of mutation [17.99 (6.1–22.54) %]. Homozygous T162I did not benefit from BS in terms of weight loss compared to their age- and sex- matched controls. Heterozygous T162I and heterozygous I170V benefited similarly from BS compared to the controls. Response to liraglutide treatment was comparable in *MC4R* T162I heterozygous patient and control. Outcomes of orlistat use was inconsistent: patient heterozygous for I170V mutation had a marginal weight gain, while age- and sex-matched wild type had over 10% excess body weight loss; homozygous T162I did not benefit and had small weight gain as much as the control. Weight loss comparison following surgical and pharmacological interventions at median 6 months between the study and control groups is shown in **Figure 4**.

Glycemic control outcomes after sleeve gastrectomy in two *MC4R*-deficient patients (heterozygous I170V and homozygous T162I) who both had T2D were substantial compared to their non-diabetic controls. On liraglutide use, the patient with heterozygous T162I had similar HbA1c lowering with his age- and sex-matched control (0.4% vs. 0.3%).

## Discussion

The melanocortin system regulates appetite and an intact leptin–melanocortin signaling is required for normal physiological response to calorie restriction (22–24). Lifestyle modification for weight loss is difficult for people with MC4R-d as they are less responsive to diet and exercise. Hence, BS is often considered as the obesity treatment of choice for individuals with MC4R-d. Our previous work has indicated that MC4R signaling may not be needed for short-term response to sleeve gastrectomy, with continuous weight loss observed several months post-BS (21). Here we report the short- and long-term outcomes of BS on weight and glycemic control in Emirati patients with *MC4R*-d, including two siblings (male and female) who had a revision of their previous LSG to a RYGB. The mutations reported in our study, both T162I and I170V, are missense mutations and result in partial (T162I) and complete (I170V) loss of function of *MC4R* gene (25, 26). A recent article reported long-term weight loss outcome on bi-allelic mutation in the *MC4R* gene in a patient who underwent sleeve gastrectomy only (27). The recent systematic review reported that most of the studies on outcomes of BS in MC4R mutation found no association of *MC4R* mutations with weight loss (20). We are reporting, for the first time to our knowledge, long-term outcomes of revisional surgery (RYGB) in both sexes with the *MC4R* null genotype. Our results suggest that longer-term response may be determined by factors other than *MC4R* genotype. These factors may include sex and gut microbiome as well as other environmental factors, as may be the case in patients with “simple obesity” (28–30).

Although the role of genetics in the case of P1 and P2 cannot be delineated, how mutation type and zygosity affect BS outcomes in these patients has been observed. Weight loss 6 months post-BS was not significantly different among patients without and with heterozygous MC4R-d, suggesting that in terms of weight reduction, patients with heterozygous T162I and I170V mutations may benefit from BS as much as MC4R-normal individuals, a finding similar to a previous report (17). Another recent study has reported no significant weight loss on a patient with compound heterozygous mutation (c.105C>A; p.Y35X, c.110A>T; p.D37V) (27). Individuals with homozygous T1621 mutation in *MC4R* and those with compound heterozygous *MC4R*-d might require multiple surgeries or continued pharmacological intervention to maintain weight loss over a longer period of time. It is also noteworthy that our younger patient had a more favorable outcome in terms of weight loss, suggesting that earlier intervention in homozygous T162I is more likely to be beneficial; increasing BMI from 12 months post-BS warrant longer-term monitoring. On glycemic control, mutation type and zygosity do not appear to influence the outcomes suggesting the significance and interplay of other factors (31, 32). Adherence to a strict diet as in the case of P2 may be required for best glycemic control regardless of BS (33). The lack of diet information on the other patients as well as physical activity profile of the cohort including the control group is a limitation of the study, which may be addressed in prospective studies.

With liraglutide treatment, we have observed that response was comparable in patients with heterozygous T1621 mutation in *MC4R* and controls (34). Liraglutide treatment for 16 weeks has been reported to result in comparable weight loss and decrease in HbA1c in patients with *MC4R*-d and controls (35, 36). However, the difference in % weight loss of BS compared to liraglutide use on individuals with heterozygous T162I may still be vast (14.2% for P5). Again, the outcome of P2’s diet program on her weight and glycemic control underscores the importance of diet and lifestyle modification on *MC4R* deficits, in addition to pharmacotherapeutic course of treatment and BS. The effect of dietary and lifestyle interventions may depend on the type of mutation (37). An additional finding here is that short-term orlistat treatment did not seem to have any beneficial effects on weight loss or glycemic control for our patient with heterozygous I170V mutation.

In conclusion, we have described here outcomes of BS with or without selected pharmacotherapy in a cohort of patients with heterozygous and homozygous *MC4R* mutations. Our data indicate beneficial but varied long-term effects of BS on these patients, which suggests that life-long monitoring and additional therapies including adjunct pharmacotherapy and revisional BS should be considered. Further elucidation of the mechanisms behind these results requires a larger cohort of patients in multicenter studies.

## Data Availability Statement

The data presented in the study are deposited in the ClinVar repository, accession numbers SCV002014765.1, SCV002014763.1, SCV002014764.1.

## Ethics Statement

The studies involving human participants were reviewed and approved by the Research Ethics Committee, Imperial College London Diabetes Centre. Written informed consent to participate in this study was provided by the participants and/or participants’ legal guardian/next of kin.

## Author Contributions

EF: study design, data acquisition and interpretation, and manuscript writing; SR: data acquisition, statistical analyses and manuscript writing; TA: study design and manuscript writing; EN: manuscript writing; NL: study design, data interpretation, and manuscript writing.

## Conflict of Interest

The authors declare that the research was conducted in the absence of any commercial or financial relationships that could be construed as a potential conflict of interest.

## Publisher’s Note

All claims expressed in this article are solely those of the authors and do not necessarily represent those of their affiliated organizations, or those of the publisher, the editors and the reviewers. Any product that may be evaluated in this article, or claim that may be made by its manufacturer, is not guaranteed or endorsed by the publisher.
